# Perspectives on digital health and advanced treatment referral in Parkinson’s care among Danish neurologists: a mixed methods study

**DOI:** 10.3389/fneur.2025.1618348

**Published:** 2025-12-10

**Authors:** N. L. C. Karottki, T. H. Thomsen, P. J. Jennum, S. Bibi, M. Sharifi, Ö. Coskun, B. Biering-Sørensen

**Affiliations:** 1 Department of Neurology, Movement Disorder and Pain Research Center, Rigshospitalet, Copenhagen, Denmark; 2 Department of Neurology, Movement Disorder Clinic, Rigshospitalet, Copenhagen, Denmark; 3 Department of Brain and Spinal Cord Injuries, Rigshospitalet Glostrup, Glostrup, Denmark; 4 Department of Health Technology, Technical University of Denmark, Copenhagen, Denmark; 5 Department of Clinical Neurophysiology, Danish Center for Sleep Medicine, Rigshospitalet, Copenhagen, Denmark; 6 Department of Public Health, Faculty of Health and Medical Science, University of Copenhagen, Copenhagen, Denmark

**Keywords:** Parkinson’s disease, digital health, wearable devices, referral criteria, mixed methods

## Abstract

**Introduction:**

Advanced treatments such as infusion therapies and deep brain stimulation can improve symptoms in Parkinson’s disease, but identifying the right patients at the right time remains challenging. Digital health technologies offer objective, continuous, and remote symptom tracking, making them increasingly relevant in Parkinson’s management. This study examines Danish neurologists’ perspectives on current referral practices for advanced Parkinson’s treatment and explores the perceived advantages and barriers of digital health technologies use in clinical decision-making.

**Methods:**

Using a mixed methods approach, we surveyed neurologists involved in Parkinson’s management across outpatient hospital settings and private practices.

**Results:**

Nineteen neurologists completed the survey, and six participated in semi-structured interviews. Most neurologists (15/19, 79%) believe current referral criteria for advanced treatment need improvement, and only (5/19, 26%) regularly use available decision-support tools.

**Discussion:**

The perceived advantages of digital health technologies include improved treatment optimization, real-world symptom tracking, and enhanced patient health literacy. However, concerns include uncertainty about the clinical relevance of measurements, resource constraints, and lack of supporting evidence. Neurologists also expressed reservations about reduced patient interaction and the insufficient tracking of non-motor symptoms in current digital health technologies. Our findings should be considered exploratory but highlight the limitations of current referral strategies for advanced treatment and neurologists’ mixed perspectives on digital health technologies, with qualitative insights revealing both optimism and concerns about implementation. Digital health technologies have the potential to aid in identifying people with Parkinson’s who may benefit from advanced treatment, and future referral criteria may benefit from incorporating objective digital measurements.

## Introduction

Healthcare systems worldwide are facing increasing strain due to aging populations and the rising prevalence of age-related chronic diseases. Among these, neurodegenerative disorders such as Parkinson’s disease (PD) pose a major challenge because of their complex and progressively disabling course. The prevalence of PD has nearly doubled over the past 25 years, affecting ~8.5 million people worldwide by 2019 and projected to affect more than 12 million people by 2040 (1, 2). PD is characterized by motor (e.g., tremor, bradykinesia, rigidity) and non-motor symptoms (e.g., sleep and autonomic disturbances), which vary substantially between individuals (3).

In Denmark, recent estimates put PD prevalence at ~200 per 100,000 adults (≈11–12 k people) (4). Although no national estimate of advanced PD prevalence is publicly available, nationwide registry data document 612 DAT initiations during 2008–2016 with marked regional variation in initiation rates (5).

In advanced stages of PD, the symptom burden increases and is complicated by fluctuations that are hard to control on oral or transdermal medication (6). Earlier reports have estimated that 38–51% of PD patients in hospital setting have advanced PD (7, 8). Inadequately treated advanced PD has a large impact on quality of life and risk of falls (9–11). Here, advanced therapies (device-assisted therapies, DAT), such as deep brain stimulation, subcutaneous apomorphine infusion, and intraduodenal or subcutaneous levodopa infusion, provide effective symptomatic treatment (12). In Denmark, initiation of all DATs is restricted to tertiary movement disorder centres, whereas private neurologists and hospital-based specialists refer patients for evaluation. Private-practice neurologists may refer patients who may be eligible, but initiation and follow-up are managed by specialised multidisciplinary teams at the tertiary centres. The centralised structure ensures that DAT candidates are evaluated by experienced clinicians, yet it may also contribute to delays or variability in referral practices across care levels. Early treatment with DAT can enable better symptom stabilisation before severe fluctuations and loss of function become difficult to reverse (13). However, identifying appropriate candidates for DAT in a timely manner is complex, particularly in the primary sector, where DAT evaluation is not part of routine neurological practice.

Existing DAT referral tools rely largely on subjective reporting and see limited use (14). While patient diaries are commonly used for tracking symptoms, they have well-known limitations such as recall bias, diary fatigue, and poor capture of dyskinesia and symptoms in advanced PD (15, 16).

Because clinic-based observations may not reflect daily life, digital health technologies (DHTs) such as wearable devices offer objective data that can bridge this gap (17–20). These rapidly evolving tools can support earlier diagnosis, better treatment monitoring, and more timely therapeutic decisions (21–23). Many people with PD (PwP) are managed by non-specialists, for whom timely DAT referral decisions can be particularly challenging. Objective data from digital health technologies (DHTs) could support this process, yet their integration into routine PD management remains limited (23–25).

Understanding how neurologists not specialized in PD identify PwP needing advanced treatment and how digital tools might support this process is essential. Although previous studies have evaluated the implementation of DHT in Parkinson’s care, none have examined neurologists’ perspectives on using such tools to support DAT-related decision-making, nor how these views vary across care levels within the Danish healthcare system (26, 27). Previous reviews have highlighted that successful digital-health implementation in PD care remains limited by organizational and behavioural factors despite the growing maturity of available technologies (28). Broader evaluations have identified similar barriers and facilitators to technology adoption across healthcare contexts (27, 29). Together, these reports underscore that clinician engagement remains central to translating digital monitoring into routine PD management. Furthermore, identifying potential barriers against the use of DHTs is important to facilitate implementation and integration.

To address these gaps, we aimed to evaluate and explore Danish neurologists’ perspectives on the use of digital health technologies in Parkinson’s care, particularly how such tools might support the identification and referral of patients for advanced treatments.

## Methods

We employed a convergent mixed-methods design with parallel data collection to capture neurologists’ perspectives on DHTs in PD care. Quantitative and qualitative data were analysed separately and later compared to identify convergent and divergent patterns, providing a more comprehensive understanding of the topic (30). A quantitative survey aimed to capture overall insights related to the target areas, i.e., referral practices and DHT, while qualitative semi-structured interviews with a nested sample offered an in-depth exploration of individual experiences, concerns, and expectations. Respondents are divided into Hospital-Based Neurologists (HBN) and Private Practice Neurologists (PPN).

### Survey

We developed a quantitative survey based on a review of existing literature and internal expert input to gather insights from neurologists working in cross-sectional settings regarding their perspectives on the use of digital data in the management of PD. The survey was developed by the study team in consultation with a public health expert, to ensure relevance across clinical settings. The survey is provided in Supplementary File, S1.

The survey comprised two sections:

### Descriptive

- Demographic information (e.g., age, gender, sector, years of experience)
- Clinical practice details (e.g., frequency of consultations, self-rated expertise, referral practices).

### Explorative

- Perspectives on the use of DHT (e.g., familiarity, perceived barriers, and potential benefits).

These sections aimed to identify current clinical practice and explore current use and opinions on DHTs. Due to the context of improving DAT referrals, we chose to focus on opinions on objective measurements from wearable devices and similar technologies. Other DHTs typically include telehealth, e-health portals, electronic health journals and apps. This was done to identify recurring patterns and illustrate differences between sectors.

Questions included 5-point Likert scales with a neutral midpoint, dichotomous yes/no items, and checklists with multiple options and free-text fields.

Data were analysed descriptively using SurveyXact and Microsoft Excel. For categorical variables, we calculated frequencies and percentages, and for continuous variables, we reported means ± standard deviations. Missing data were handled on an item-by-item basis without imputation. Given the small and exploratory sample, analyses were descriptive and not inferential. Differences between groups were interpreted qualitatively.

### Participants and distribution

Invitation emails were sent to all neurologists managing people with Parkinson’s disease in Denmark, including those working in 13 hospital-based movement-disorder clinics (approximately 24 neurologists) and 38 private practices, for a total of about 63 eligible clinicians. The survey was distributed electronically via SurveyXact, and responses were collected over 3 months with reminder emails at four and 8 weeks. Participation was voluntary, confidential, and anonymous. The study targeted all neurologists with regular involvement in Parkinson’s management across hospital and private-practice settings.

### Semi-structured interview

To further elucidate neurologists’ opinions on DHT in PD care, we conducted semi-structured interviews. Initially, we developed an interview guide based on existing literature, clinical experience, and questions included in our survey, to ensure consistency across interviews while allowing for flexibility in responses (Supplementary File, S2).

Participants were neurologists working with PwP in both tertiary movement-disorder clinics and private practice, recruited through professional networks to ensure diversity in age, gender, clinical experience, and familiarity with DHTs. Six neurologists (three from hospital settings, three from private practice, age range 34–68, balanced gender distribution) participated. Interviews were conducted by co-authors SB, MS, and OC, who underwent preparatory training and test interviews to standardise their approach. None of the interviewers had prior acquaintance with participants. Each interview lasted approximately 30 min and was conducted either in person or online during the survey period. Data collection continued until no new major topics emerged.

All interviews were audio recorded and transcribed verbatim. We systematically read the transcripts, assigning initial codes to segments and grouping repeating concepts into basic themes. Codes were derived inductively following Braun and Clarke’s framework and refined through investigator triangulation (31). From these basic themes, we performed a thematic network analysis by categorizing concepts further into three subthemes and one global theme, based on Attride-Stirling (32).

To ensure the trustworthiness of the data analysis, the first author independently reviewed the final thematic groupings, and sought clarification and consensus in a triangulation process with the research group. A figure of the thematic network analysis is provided in Supplementary File, S3.

The Danish Medical Research Ethics Committee determined that the study did not require formal approval as it did not involve patients or sensitive personal data. All participating neurologists received written information about the study and provided informed consent before participation.

## Results

### Survey

#### Demographics and clinical practices

This section provides insights into neurologists’ demographics and clinical practices, with a focus on their experience in managing PD and frequency of consultations. Key differences between HBNs and PPNs are separated into two columns in Table 1.

**Table 1:** Descriptive data of participating neurologists.

	Hospital-Based Neurologists (*n* = 10)	Private Practice Neurologists (*n* = 9)
Gender, n (%)		
Men	8 (80%)	5 (56%)
Women	2 (20%)	3 (33%)
Other/not specified	0	1 (11%)
Age (years), mean ± SD	49.8 ± 9.5	55.1 ± 7.4
PD experience (years), mean ± SD	14.2 ± 11.2	11.8 ± 8.5
Self-rated as specialized in PD [n, (%)]	9 (90%)	3 (66%)
Frequency of consultations for PwP (n (%), every 6 months or more often)	2 (20%)	9 (100%)
Average number of PwP referred per year for advanced treatment (n)	12.4^*^ ± 11.4	8.4 ± 7.0
Use of decision-support tools (n, %)^**^	4 (40%)	1 (11%)

^*^Includes neurologists working in clinics that provide advanced treatment. ^**^Decision-support tools include items like 5-2-1 criteria or similar available tools (14). PD, Parkinson’s disease; PwP, Person with Parkinson’s disease; SD, Standard Deviation.

Of the 63 invited neurologists, 19 completed the survey (response rate ≈ 30%), including 10 hospital-based and 9 private-practice neurologists. This sample represents roughly one-third of Danish neurologists routinely involved in Parkinson’s care. These subgroup differences were interpreted descriptively and should be regarded as exploratory due to the small sample size.

PPNs were, on average, slightly older (age 55.1 ± 7.4 years vs. 49.8 ± 9.5 years for HBNs) and had slightly fewer years of experience managing PwP (11.8 ± 8.5 years vs. 14.2 ± 11.2 years). Moreover, a higher percentage of hospital neurologists evaluated themselves as specialized in movement disorders/PD (90% vs. 66%).

The frequency of consultations was higher for PPNs, with 100% of their PwP having consultations every 6 months or more often. For hospital clinics, 20% of PwP have consultations every 6 months or more often, 70% every six to 12 months and 10% see their neurologist/PD nurse less frequently than every 12 months.

#### Referral practices

**Figure 1:**
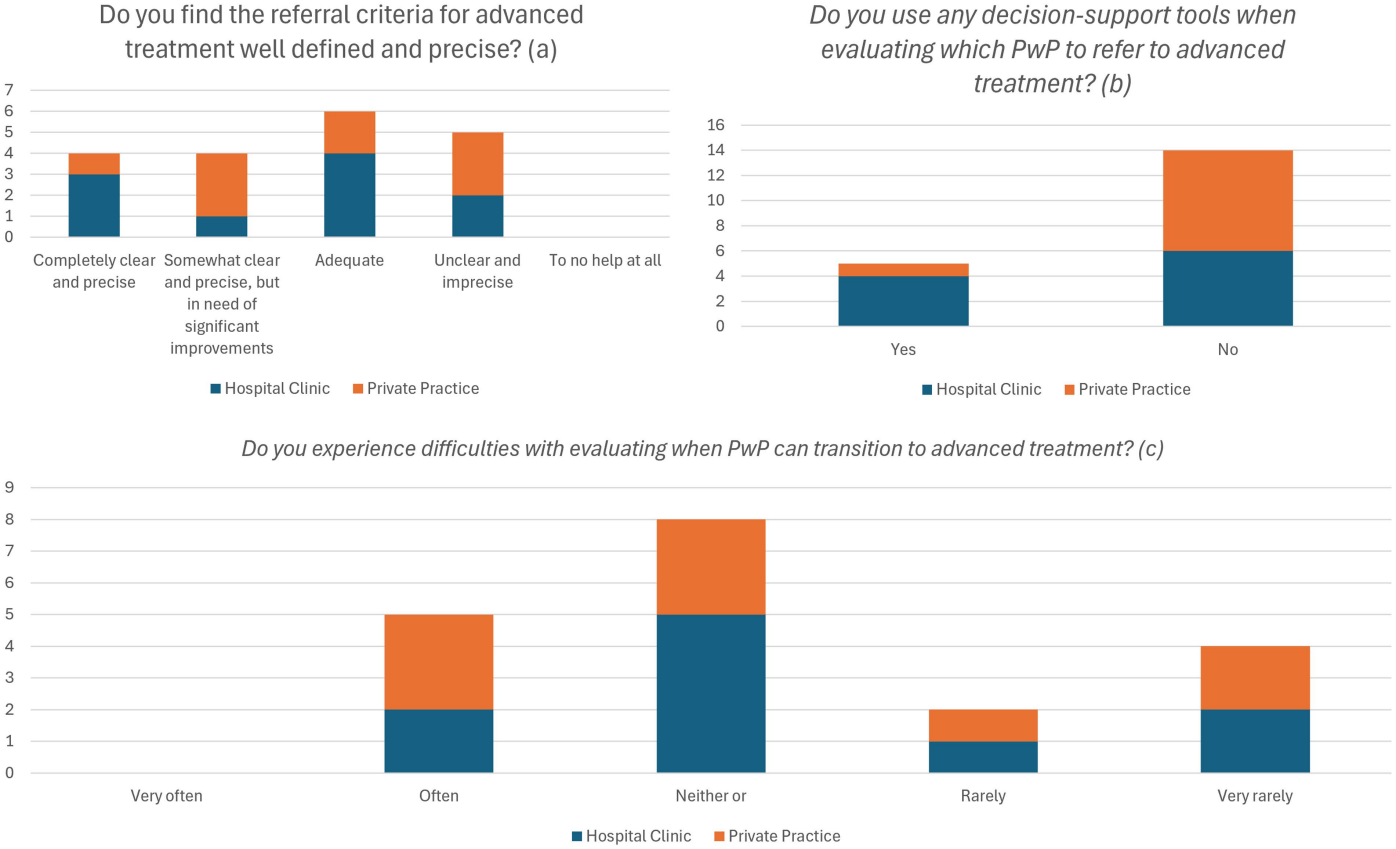
Overview of opinions on referral standards **(a)**, use of decision-support tools for DAT (Device-Assisted Therapy) **(b)**, and current referral practices **(c)**. Data is presented as frequencies of responses. PD, Parkinson’s disease; PwP, Person with Parkinson’s disease.

21% of respondents (3 HBNs and 1 PPN) considered the current referral standards for advanced treatment to be completely clear and precise (Figure 1a). 40% (*n* = 4) of HBNs and 11% (*n* = 1) of PPNs used available decision-support tools for referral to DAT (Figure 1b). A minority (32%, *n* = 6) reported that they ‘rarely’ or ‘very rarely’ experience difficulties in choosing the appropriate time for PwP to be referred to DAT (Figure 1c).

**Figure 2:**
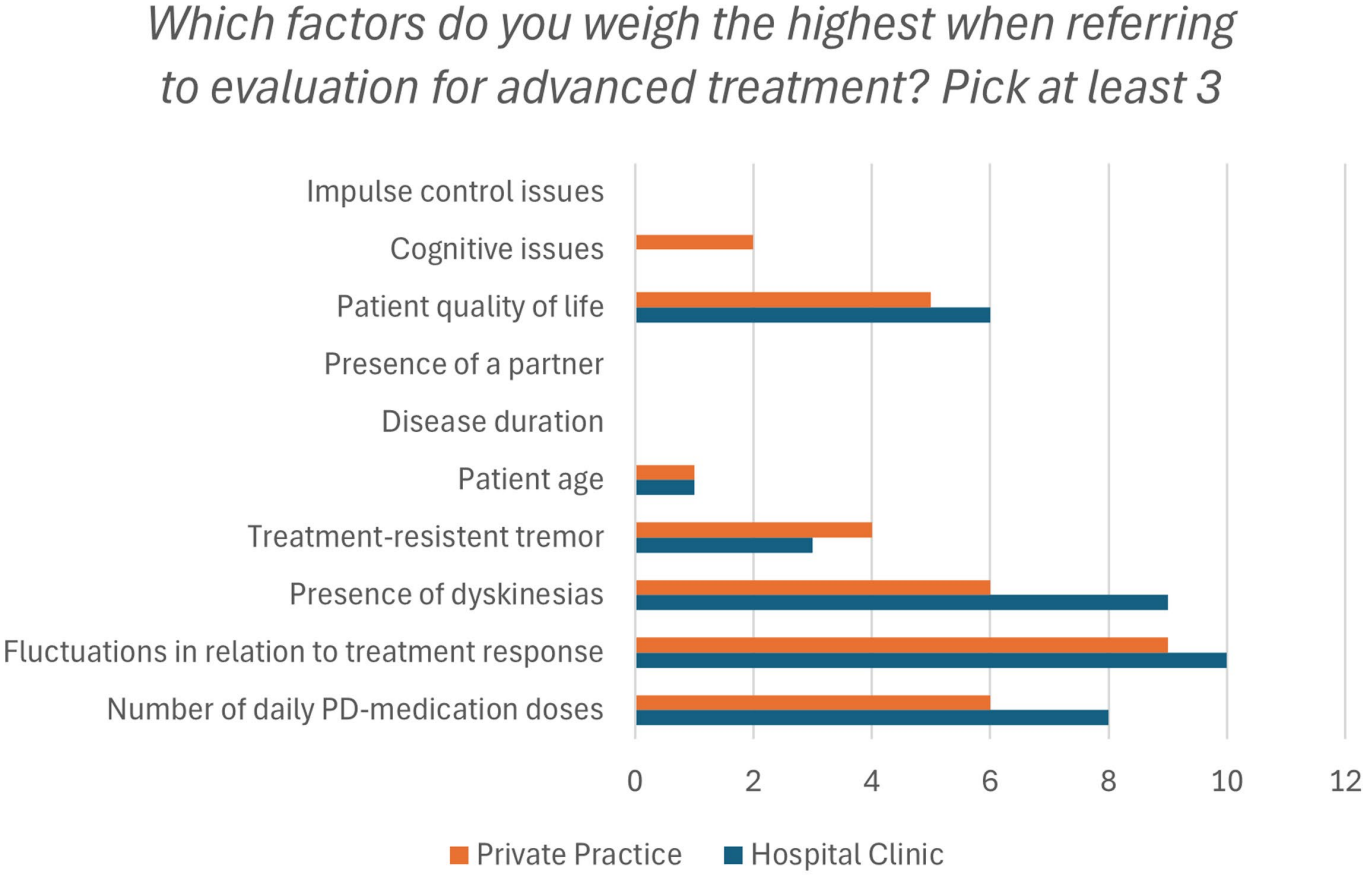
Factors that affect the evaluation of DAT (Device-Assisted Therapy) eligibility. Presented as frequencies. PD, Parkinson’s disease.

When asked which factors contributed the most to DAT referrals, both groups emphasized symptom fluctuations, dyskinesias, and number of daily medication dosages (Figure 2).

#### Perspectives on digital health technologies

Most respondents (52.6%, *n* = 10) rated the potential of digital objective measurements as average, while 26.3% (*n* = 5) rated it positively, and 21.1% (*n* = 4) rated it as poor. HBNs had more experience with tools such as wearable sensors and mobile apps (60%, *n* = 6 and 40%, *n* = 4, respectively) compared to PPNs (11% for both tools, *n* = 1).

Most respondents had experience with DHTs though 21.1% (*n* = 4) had never used any. HBNs had experience with video consultations, wearable sensors, and mobile apps, while PPNs primarily used video consultations. When asked if they would use digital objective measurements as part of their decision-making for managing PwP if integrated into daily clinical practice, 84.2% (*n* = 16) of respondents answered yes. Furthermore, 78.9% (*n* = 15) of respondents stated that they would be motivated to participate in workshops or other educational activities focused on improving digital skills in relation to PD management.

**Figure 3:**
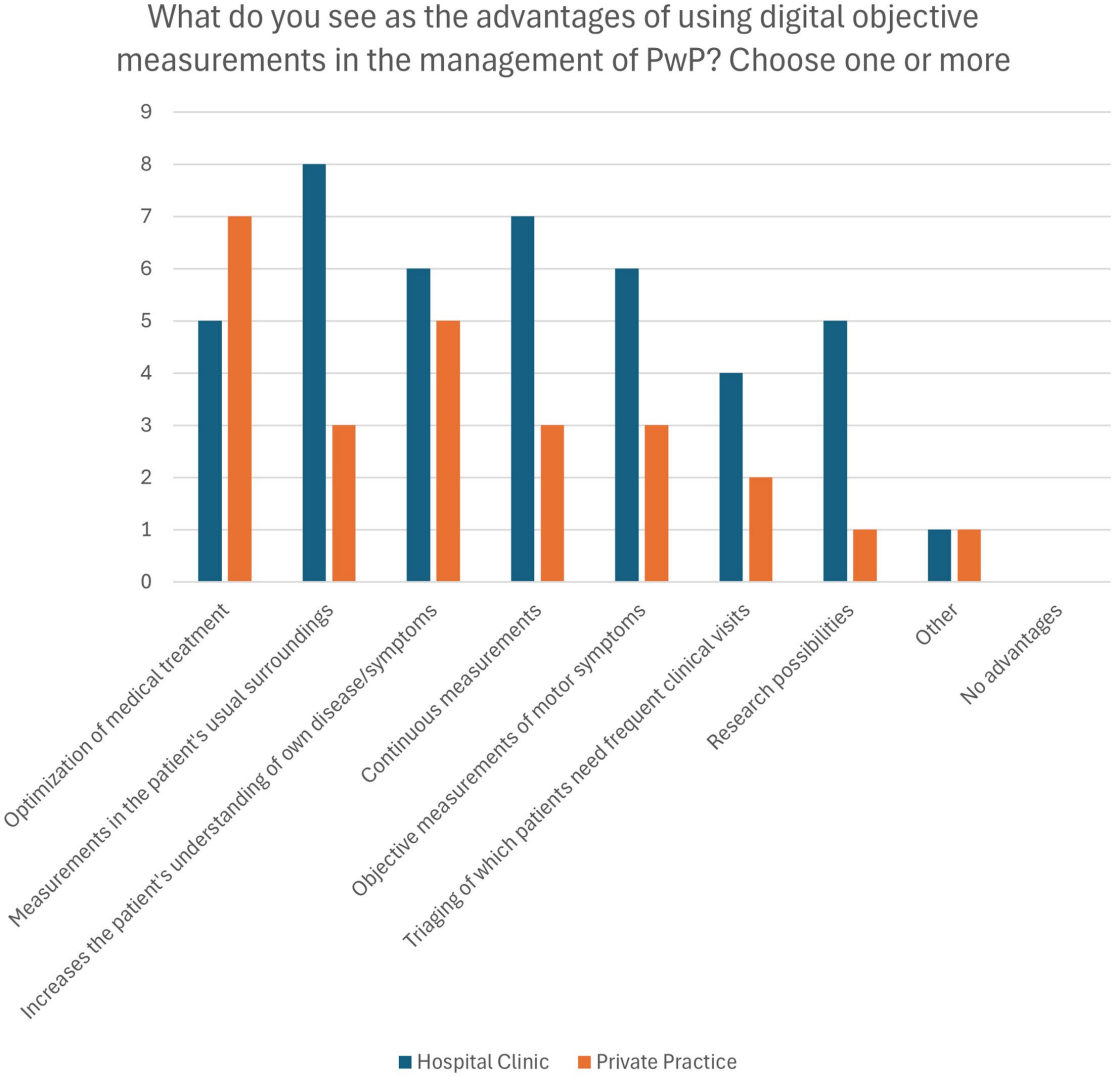
Perceived advantages of using Digital Health Technologies in Parkinson’s disease management. Presented as frequency of responses.

The primary advantage of DHTs cited by respondents was the optimization of medical treatment (63.2%, *n* = 12, Figure 3). HBNs were more likely to emphasize the benefit of collecting data in patients’ usual living environments (80%, *n* = 8) compared to PPNs (33%, *n* = 3). 57.9% (*n* = 11) of both groups noted that digital measurements can increase patients’ understanding of their own disease and symptoms. 22% (*n* = 2) of PPNs saw no advantages in the implementation of digital health solutions, specifying lack of necessary digital infrastructure and digital competencies in low population areas as the reason.

**Figure 4:**
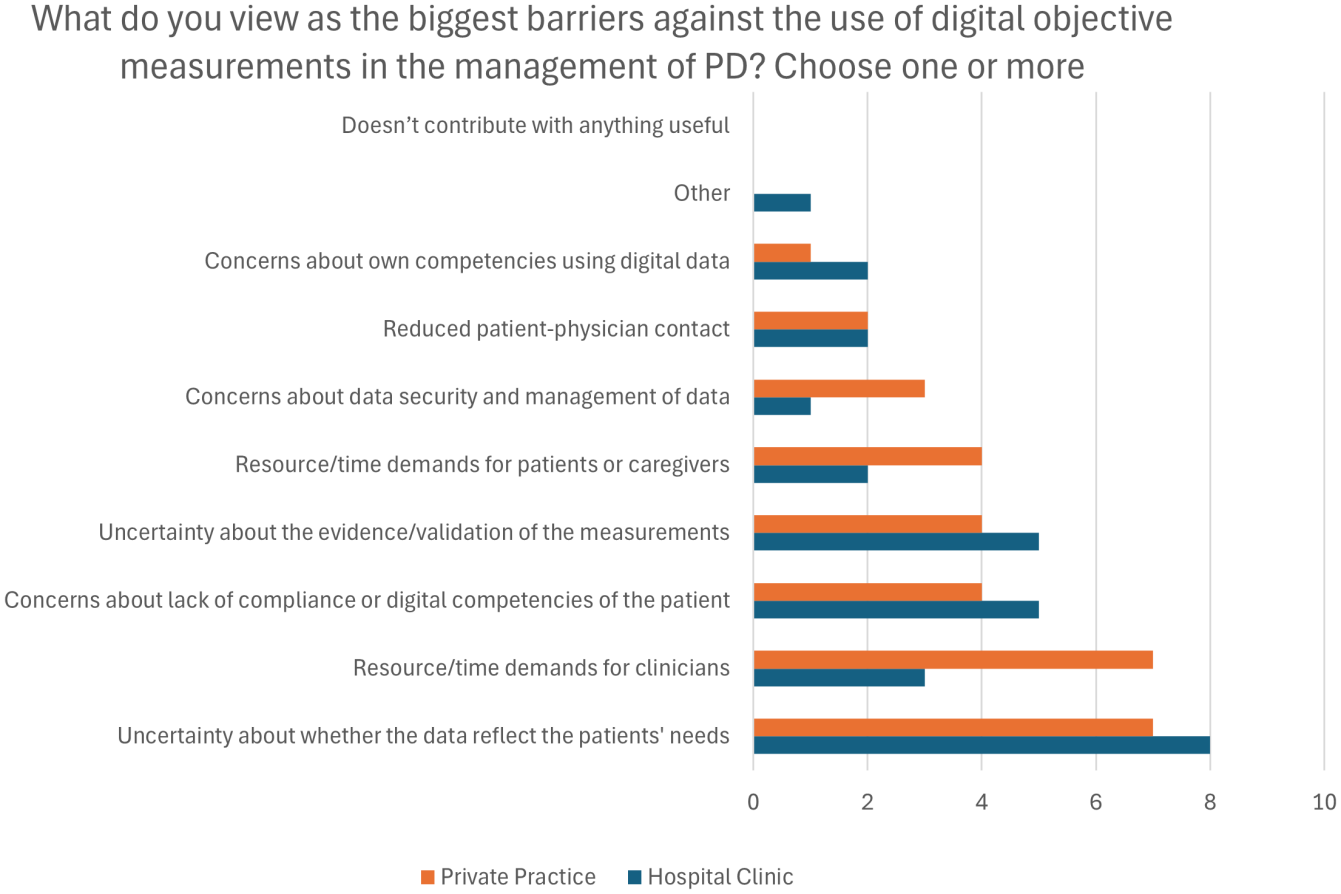
Perceived barriers against the use of objective measurements from Digital Health Technologies. Presented as frequency of responses. PD, Parkinson’s disease.

The most significant barrier, reported by 78.9% (*n* = 15) of the respondents in both groups, was uncertainty about whether digital measurements reflect the patients’ needs (Figure 4). PPNs were particularly concerned about time and resource demands using DHTs (78%, *n* = 7 vs. 30%, *n* = 3 of hospital neurologists). Other notable barriers included concerns about the evidence supporting the validity of the measurements, and patient compliance and digital competencies (47.4%, *n* = 9 of respondents).

### Semi-structured interviews

Upon analysing and coding the transcripts from six semi-structured interviews, we conducted a thematic network analysis that resulted in three primary subthemes: (1) perspectives on the potential of digital health technologies (DHTs), (2) concerns regarding their use, and (3) challenges associated with digitalised patient contact. The overarching global theme was identified as *the influence of digitalisation on neurological practice*.

We selected representative quotations to illustrate each subtheme and indicated the responder type as hospital-based neurologist (HBN) or private-practice neurologist (PPN).

#### Perceived potential of digital health technologies

In our interviews, neurologists from both groups emphasised how DHTs could improve symptom reporting, help prepare in advance, and focus patient care on specific issues. One neurologist explained, *“I think [DHTs] could help me, because I sometimes get frustrated about the lack of reasonable answers… Especially in the cases where answers are pointing in all directions, and you are very much in doubt about whether the information you are getting is true. Then you end up medicating based on a theory about the patient, that may not reflect reality”* (PPN). Another highlighted the advantage of having pre-consultation data available, stating, *“Data needs to be available before consultations… so we can focus on the areas where the patient has issues”* (PPN).

In the context of referrals for advanced treatment, one hospital-based neurologist elaborated: *“Infusion pump therapy is very expensive… and the Region may decide that they need some kind of objective documentation for fluctuations before you can start that type of treatment”* (HBN). Overall, participants expressed cautious optimism about digitalisation, summarised by one private-practice neurologist: *“I’m positive about it - it’s a good opportunity if we use it the right way”* (PPN).

#### Concerns regarding digital health technologies

Despite overall optimism, neurologists expressed reservations about the clinical reliability and practical implications of digital health technologies. Concerns were primarily raised by HBNs, who had greater experience with advanced treatment and exposure to DHTs in clinical research. Several participants questioned the robustness of device-generated data, describing a need for stronger evidence before adoption. One hospital-based neurologist explained, *“I trust colleagues who have more experience, but I need to see solid evidence before I rely on the data”* (HBN). Another added, *“I mainly know which research projects are testing these devices, but I’m still not sure how reliable they really are”* (HBN).

Some also feared that growing reliance on quantitative data might distort the clinical encounter. As one neurologist noted, *“We risk focusing on the numbers and losing sight of the patient in front of us”* (HBN). These concerns reflected a broader hesitation to integrate DHTs before their validity, interpretation, and clinical relevance are fully established.

#### Challenges associated with the digitalized patient contact

Both hospital-based and private-practice neurologists described challenges related to maintaining patient-centred care as clinical practice becomes increasingly digitalised. Several participants were concerned that digital tools might shift focus from people to data, as one neurologist emphasised: *“We aren’t treating data; we are treating people”* (PPN). Another echoed this sentiment, asking, *“Patients do not necessarily agree with the data, and what do you do then? Treat the data or treat what the patient reports?”* (HBN).

Participants also noted that current DHTs capture only part of the clinical picture. As one neurologist observed, *“Parkinson’s disease is largely a non-motor disorder, and you cannot capture depression, fatigue, and pain with wearables”* (HBN). Others pointed out that contextual factors such as infections or social stressors remain invisible to digital systems.

Finally, several neurologists questioned whether all patient groups would benefit equally. One explained, *“When we get access to new technology, we first and foremost need to think about where it is relevant… we can use it on all our patients, but is it really relevant?”* (HBN). These reflections highlight that while DHTs may enrich patient monitoring, they risk narrowing clinical attention and must be applied judiciously to maintain a human-centred approach.

#### Integration of findings

Quantitative and qualitative findings showed clear areas of convergence, particularly regarding barriers to DHT adoption and the perceived clinical value of objective measurements. Minor differences were limited to perceptions of workflow feasibility. An overview of the integrated findings is provided in Table 2.

**Table 2:** Thematic matrix showing convergence between survey and interview findings in a mixed-methods analysis.

Theme	Survey	Interviews
Clarity and timing of DAT referrals	Only 21% rated current referral standards as completely clear and precise. About one third reported they rarely or very rarely struggle with timing of referral.	Neurologists described grey zones and uncertainty about when to refer, especially outside tertiary centres. They expressed a need for clearer shared guidance.
Setting differences and collaboration	Use of decision-support tools was higher among hospital-based neurologists (40%) than private practitioners (11%). There were differences in consultation frequency and self-rated specialization.	Private-practice clinicians reported weak feedback loops with tertiary centres and unclear responsibilities. They requested better-defined referral pathways.
Perceived value of digital health technologies (DHTs)	Most respondents (84%) would use objective digital measurements if they were integrated in routine care. Many highlighted treatment optimization and improved patient understanding as main benefits.	Interviewees viewed DHTs as useful for documenting fluctuations, preparing for visits, and supporting communication with tertiary teams.
Barriers to DHT adoption	Main barriers included uncertainty about whether measures reflect patients’ needs (79%), lack of time and resources (more common among private neurologists), and concerns about evidence and compliance.	Participants called for stronger validation, simple reporting formats, and training. They worried about workload and unclear division of responsibility for data interpretation.
Patient–clinician interaction	Some neurologists feared DHTs could reduce personal contact with patients.	Interviewees stressed that technology must not replace dialogue, summarised by the quote “We are not treating data, we are treating people.”
Readiness and education	About 79% indicated interest in training to improve digital skills. Hospital-based neurologists had more experience with wearables and mobile apps than private practitioners.	Interviewees expressed cautious optimism. Those with prior exposure to DHTs were most enthusiastic and requested brief, practical training opportunities.

Themes reflect Danish neurologists’ perspectives on device-assisted therapy (DAT) referral practices and digital health technology (DHT) use in Parkinson’s disease care.

## Discussion

Only one-third (32%, *n* = 6) of neurologists in our survey reported rarely or very rarely having difficulty evaluating a patient’s readiness for advanced treatment. Furthermore, only 30% (*n* = 3) of HBNs and 11% (n = 1) of PPNs found current methods of DAT referral to be clear and precise. Similar challenges were highlighted in a recent Dutch survey study, where the authors found that 44% of respondents (HBNs) found DAT referral criteria to be clear and 47% reported that they had the necessary expertise to evaluate eligibility for DAT (33). PPNs and general practitioners (i.e., non-specialists), who are less likely to encounter advanced treatment scenarios compared to their hospital counterparts, are especially impacted by the lack of well-defined criteria (34, 35). Combined with the underutilization of referral tools we observed, the need for accurate and user-friendly criteria continues to be ever relevant.

In our survey, symptom fluctuations were reported as the main indication for DAT, an area in which having access to remote, objective, and continuous data is particularly well suited. In our interviews, three neurologists emphasized the importance of accessing fluctuation measurements, particularly in the context of DAT. One HBN even envisioned a future where policymakers might mandate objective data before approving funding for costly DATs.

Despite most survey respondents acknowledging the potential value of digital objective measurements gained from DHTs, wearable devices and mobile apps are mainly confined to hospital settings, limiting experience with these tools in daily clinical practice. The cause is likely to be barriers mentioned both in the survey responses and interviews: perceptions of high resource and time demand, and a lack of clear clinical evidence supporting their use. The cost-effectiveness of DHT remains debated, particularly due to the lack of solid evidence for clinical benefit (36, 37). Several validation studies have been made, but evidence of impact on quality of life is lacking (38). These uncertainties mirror findings from recent implementation studies, which emphasize that, although regulatory-approved digital tools now exist for PD monitoring, their uptake depends on workflow integration and perceived clinical utility (27, 28).

In our survey, we focused on objective measurements gathered from digital devices and thus did not include telemedicine or social media in our analyses.

The main recurring barriers in our study are uncertainty about validity of the measurements, and whether the measurements reflect the patients’ needs. This scepticism was particularly marked among neurologists who lacked consistent access to advanced digital tools, contributing to hesitancy in integrating them into routine clinical practice.

Across several diseases, evidence for the clinical value of symptom tracking is limited, as most existing trials are either too small, lack long-term follow-up, or fail to assess meaningful patient-centred outcomes, such as quality of life (39, 40). This lack of robust evidence not only limits clinician confidence in objective symptom tracking but also hinders the establishment of standardized practices for integrating digital health solutions into routine care. Most sensor technologies in PD primarily focus on motor symptoms, despite increasing recognition of the importance of non-motor symptoms – something the neurologists in our study found as a major flaw. Digital biomarkers for non-motor symptoms are gaining attention but remain underutilized in most existing devices (22).

Survey respondents also raised concerns about impaired patient-physician interaction, suggesting a fear that digital tools could potentially interfere with the quality of patient care. This is further supported in the qualitative interview: *“We aren’t treating data; we are treating people*,” reflecting a broader apprehension about whether DHT might detract from the human aspect of care, where patient trust and direct communication are crucial. Overreliance on DHTs and how they may be used (or abused) by clinicians less familiar with PD management may be a major pitfall (41). As such, there appears to be a dichotomy of DHTs providing more personalized care but also posing a risk for the care being too focused on the specific niches captured. It remains highly relevant to identify the right patients to utilize DHTs for, as mentioned by three neurologists in our interviews. Opinions in the literature on the impact DHTs will have on daily clinical practice vary greatly, but the tendency appears to be in support of DHTs facilitating changes (not purely positive or negative) in the patient-physician dynamic, shifting focus to shared decision-making (42–46). Comparable patterns have been reported in other neurological populations, where patients expressed interest in remote monitoring yet highlighted usability and trust as key determinants of adoption (47).

Nearly half of the neurologists in our survey (47%) expressed concerns about patients’ ability to effectively use digital products due to cognitive decline. Moreover, one neurologist highlighted a lack of digital health literacy among healthcare providers themselves, stemming from limited exposure to digital products. Digital health literacy for PwP is a significant concern in the increasingly digitalized PD landscape, as recently explored (48). While no studies specifically address digital health literacy for PwP, estimates suggest that up to 30% of PwP have low health literacy, and 21% lack basic digital skills. In contrast to the otherwise valid point raised by the neurologists, digital skills of PwP do not appear to differ significantly from those observed in a neurologically healthy, age-matched population (49). In fact, while limited digital competencies may be a barrier, PwP have shown an eagerness to incorporate DHT into their disease management (41, 50). A recent study showed willingness to integrate remote monitoring and DHTs amongst both PwP and neurologists, based on data from a Finish and Italian population (51). This is reflected in our data, where almost all respondents find potential benefits from the implementation of DHTs. Moreover, the neurologists are ready to prioritize time to be educated in the use of DHTs if offered.

Overall, our findings indicate that most neurologists perceive current referral criteria as in need of improvement, with variability in referral practices between HBNs and PPNs. Currently available decision-support tools for referral are barely utilized, especially by PPNs. We further highlight the variability in the adoption of digital tools among neurologists, with qualitative insights revealing both optimism and perceived barriers to implementation. Most neurologists in our study recognize the potential for DHTs to optimize PD care if the digital infrastructure supports it and educational programs are provided.

### Limitations

This study has several limitations. The survey sample was small and obtained via non-probability recruitment, which limits generalizability and invites selection and coverage bias. The achieved participation (~30%) is slightly lower than typical for web-based clinician surveys, and the mixed-methods design helps triangulate convergent signals across methods (52). Accordingly, our results should be viewed as hypothesis-generating and context-specific to Denmark’s centralized DAT pathway. The inductive coding approach used for qualitative analysis is interpretive and not easily scalable, although triangulation among multiple researchers helped reduce subjectivity. As the study was conducted within the Danish healthcare context, transferability to other settings may be limited. Finally, while neurologists’ perspectives were examined in depth, patient viewpoints and non-motor symptom domains were not included and should be explored in future research.

### Conclusion

Danish neurologists acknowledge the limitations of current referral strategies for device-assisted therapies and express cautious optimism about the role of digital health technologies in Parkinson’s care. While digital tools may help identify suitable candidates for advanced treatment and support more informed decision-making, their value depends on clinical validation, adequate infrastructure, and seamless integration into clinical workflows. The findings highlight the need for targeted education, shared digital frameworks between hospital and private sectors, and the inclusion of non-motor and patient-centred outcomes in future developments. Broader, larger, and more diverse studies are warranted to confirm these observations and to guide the responsible implementation of digital health technologies in neurological practice. In practical terms, short-term priorities include clinician training, optimization of workflow integration, and establishing consensus on how to interpret continuous digital data in routine visits. Longer-term goals should focus on validation studies in real-world settings, sustainable funding structures for device deployment, and the expansion of digital measures to capture non-motor symptoms. Collectively, these results complement recent mixed-methods work in primary-care and specialist settings and reinforce that context-specific factors must be considered when planning digital-health implementation.

## Data Availability

The raw data supporting the conclusions of this article will be made available by the authors, without undue reservation.
