# Supplementary material for: Perspectives on digital health and advanced treatment referral in Parkinson’s care among Danish neurologists: a mixed methods study
Source: Front Neurol. 2025 Dec 10;16:1618348. doi: 10.3389/fneur.2025.1618348 (PMC12727555; doi:10.3389/fneur.2025.1618348)
Supplement: Supplementary file 1 [file Data_Sheet_1.PDF]

Interview Guide

Introduction

Remember consent!

Who are we?
- Briefly introduce the educational background and the technologies we have insights into.

Brief presentation of the project:
In our project, we aim to investigate the attitudes, experiences, and expectations of general and hospital-based neurologists regarding digital technologies that support the treatment process with patients. The goal is to understand the opportunities and challenges seen with increased digitalization of patient contact.

Why you as an informant?
- Briefly explain how the interviewee can contribute to the project.

Themes / Research Questions & Interview Questions

Experiences:

How have neurologists' experiences with digital technologies affected their workflows and treatment processes with patients?

1. Do you have any experience with digital technology where the patient actively helps generate data?

Follow-up:
- If yes:
  - Can you give examples of the types of technologies you have experience with? (e.g., wearables)
  - Can you give examples of technology that one of your patients uses?
  - How did the process of getting the patient involved in using the technology start?

- If no:
  - What knowledge do you have of these technologies from, for example, conversations with colleagues or conferences?

2. What is the reason you use / do not use digital technology where the patient is a co-producer of data?
- If not using:
  - Is it because you are not familiar with these technologies, or was it an active choice for another reason?

3. How do you think your workflows have changed / have your workflows changed when the patient is involved in generating data for their treatment process?
Follow-up:
- Do you experience that patients use apps or wearables on their own initiative, or is it something you suggest?
- Are the consultations more structured when they bring their own data?

Attitudes:

How do neurologists' attitudes toward digital technology relate to their professional identity and their desire to maintain it?

1. What is your view on the use of digital technologies that patients can use to generate data in your clinical practice?
Follow-up:
- Have your experiences influenced whether you see it positively or negatively?

2. What advantages do you see in using digital technologies, such as wearables, compared to traditional paper-based methods?
Follow-up:
- What disadvantages do you see in using digital technologies, such as wearables, compared to traditional paper-based methods?

3. In what ways do you think digital technologies influence your autonomy as a neurologist?
Follow-up:
- Do these technologies change how you make decisions in your practice?
- Are consultations more focused?
- Are consultations more time-consuming due to your interpretation of data?
- Do the data allow you to see patients only when necessary, instead of during arbitrary annual check-ups?

Expectations:

What are neurologists' expectations regarding the potential opportunities and challenges associated with increased digitalization of patient contact?

1. We know that there are digital solutions validated for neurological patients. Does it matter to you whether a solution is validated or not?
Follow-up:
- Now that there are validated solutions, why do you think not many neurologists are using them?

2. What obstacles do you think might occur when implementing digital technologies while working with the patient during the treatment process?
Follow-up:
- Can you mention cognitive symptoms from your patients that increased digitalization of patient contact would not be able to address?

3. What are your own expectations for increased digitalization of patient contact in neurological practice?

Conclusion

Final question
Do you have anything else you would like to add regarding your attitudes, experiences, and expectations for the use of digital technologies in neurological practice?

Repeat the purpose of the interview
This interview will be transcribed to be used for thematic analysis. Of course, you will be anonymized in the transcription.

Summary
Thank you very much for your time! You will receive the project if you wish.
