# Supplementary material for: Perspectives on digital health and advanced treatment referral in Parkinson’s care among Danish neurologists: a mixed methods study
Source: Front Neurol. 2025 Dec 10;16:1618348. doi: 10.3389/fneur.2025.1618348 (PMC12727555; doi:10.3389/fneur.2025.1618348)
Supplement: Supplementary file 2 [file Data_Sheet_2.PDF]

What age are you?

_____

What is your gender?

(1) ❍ Male

(2) ❍ Female

(3) ❍ Other

(4) ❍ Prefer to not disclose

Which sector do you work in?

(1) ❍ Outpatient hospital clinic

(2) ❍ Private practice

In total, how many years have you worked with Parkinson patients?

_____

Would you categorize yourself as a movement disorder/Parkinson specialist?

(1) ❍ Yes

(2) ❍ No

How many of your weekly consultations are related to Parkinson's Disease?

(1) ❍ <10%

(3) ❍ 10-30%

(4) ❍ 31-50%

(7) ❍ >50%

How would you rate your clinical expertise in relation to management of patients with Parkinson's Disease?

(1) ❍ Very poor

(2) ❍ Poor

(3) ❍ Average

(4) ❍ Good

(5) ❍ Very good

What is the average time interval between clinical visits (with a physician) for a Parkinson patient in your clinic?

(1) ❍ Every 3 months or more often

(2) ❍ Between 3 and 6 months

(3) ❍ Between 6 and 12 months

(4) ❍ More than 12 months

How many Parkinson patients do you on average refer to evaluation for advanced treatment on a yearly basis?

_____

How many Parkinson patients do you in average refer to second opinion on a yearly basis?

_____

Do you find the referral criteria for advanced treatment well defined and precise?

(1) ❍ Completely clear and precise

(2) ❍ Somewhat clear and precise, but in need of significant improvements

(3) ❍ Adequate

(4) ❍ Unclear and imprecise

(5) ❍ To no help at all

Do you experience difficulties with evaluating when Parkinson patients potentially can transition to advanced treatment?

(1) ❍ Very often

(2) ❍ Often

(3) ❍ Neither or

(4) ❍ Rarely

(5) ❍ Very rarely

Which factors do you weigh the highest when referring to advanced treatment? Pick at least 3.

(1) ❑ Number of daily PD-medication doses

(6) ❑ Fluctuations in relation to treatment response

(2) ❑ Presence of dyskinesias

(5) ❑ Treatment-resistant tremor

(9) ❑ Patient age

(10) ❑ Disease duration

(3) ❑ The presence of a partner

(4) ❑ Patient quality of life

(7) ❑ Cognitive issues

(8) ❑ Impulse control issues

Do you use any decision-support tools when evaluating which Parkinson patients to refer to advanced treatment?

(1) ❍ Yes

(2) ❍ No

Which decision-support tools?

(1) ❑ MANAGE-PD

(2) ❑ 5-2-1 criteria

(3) ❑ ScandModis criteria

(4) ❑ D-DAT criteria

(5) ❑ Other _____

(6) ❑ None

Application of Digital Objective Measurements (DOMs)

Digital objective measurements involve the use of sensors for passive (without interaction/specific exercises) and active (e.g., during specific motor tests) monitoring of the patient's motor symptoms. The goal is to utilize these objective measurements as surrogate markers for the patient's clinical condition.

Do you have experience with the use of digital solutions in the context of patient management? Choose one or more.

(1) ❑ Wearable sensors

(2) ❑ Mobile apps

(3) ❑ ePRO

(6) ❑ Videoconsultations

(4) ❑ Other _____

(5) ❑ None

How would you evaluate the potential of using digital objective measurements to improve the management of Parkinsons patients?

(1) ❍ Very poor

(2) ❍ Poor

(3) ❍ Average

(4) ❍ Good

(5) ❍ Very good

Would you use digital objective measurements as part of your decisionmaking for the management of Parkinson patients, if it was integrated in daily clinical practice?

(1) ❍ Yes

(2) ❍ No

What do you see as the advantages of using digital objective measurements in the management of Parkinson patients? Choose one or more.

(9) ❑ Optimization of medical treatment

(1) ❑ Measurements in the patients usual surroundings

(2) ❑ Continuous measurements

(7) ❑ Increases the patients understanding of own disease/symptom

(4) ❑ Triaging of which patients need clinical visits more often

(5) ❑ Objective measurements for motor symptoms

(6) ❑ Research possibilties based on objective measurements

(8) ❑ Other _____

(10) ❑ No advantages

What do you view as the biggest challenges or barriers for the use of digital objective measurements in the management of Parkinson patients? Choose one or more.

(1) ❑ Reduced patient-physician contact

(2) ❑ Ressource/time demands for clinicians

(3) ❑ Ressource/time demands for patients or caregivers

(4) ❑ Uncertainty about the evidence/validity of the measurements

(5) ❑ Uncertainty about whether the measurements reflect the patients needs

(10) ❑ Concerns about data security and management of data

(6) ❑ Concerns about lack of compliance or digital competencies of the patient

(7) ❑ Concerns about own competencies to use digital data

(9) ❑ Doesn't contribute with anything useful

(8) ❑ Other _____

What does the digital solution need to include of functions for you to use it in your daily clinical practice? Choose one or more.

(1) ❑ User-friendliness

(3) ❑ Clear and simple overview of data (visual representation in graphs)

(4) ❑ Real-time data

(5) ❑ Patient portal for self-reporting symptoms

(6) ❑ Adequate instructions in the use of data from digital objective measurements (for clinicians and patients/caregivers)

(7) ❑ Easy access to support

(8) ❑ The possibility of using data in research

(9) ❑ Other _____

Are you motivated to participate in workshops or other educational programs with focus on improving your digital competencies in relation to management of Parkinson patients?

(1) ❍ Yes _____

(2) ❍ No

Do you have any further comments or questions about the use of digital objective measurements, including suggestions for effective use or expectations?

____

____

____
