# Supplementary figures and images for: Perspectives on digital health and advanced treatment referral in Parkinson’s care among Danish neurologists: a mixed methods study
Source: Front Neurol. 2025 Dec 10;16:1618348. doi: 10.3389/fneur.2025.1618348 (PMC12727555; doi:10.3389/fneur.2025.1618348)

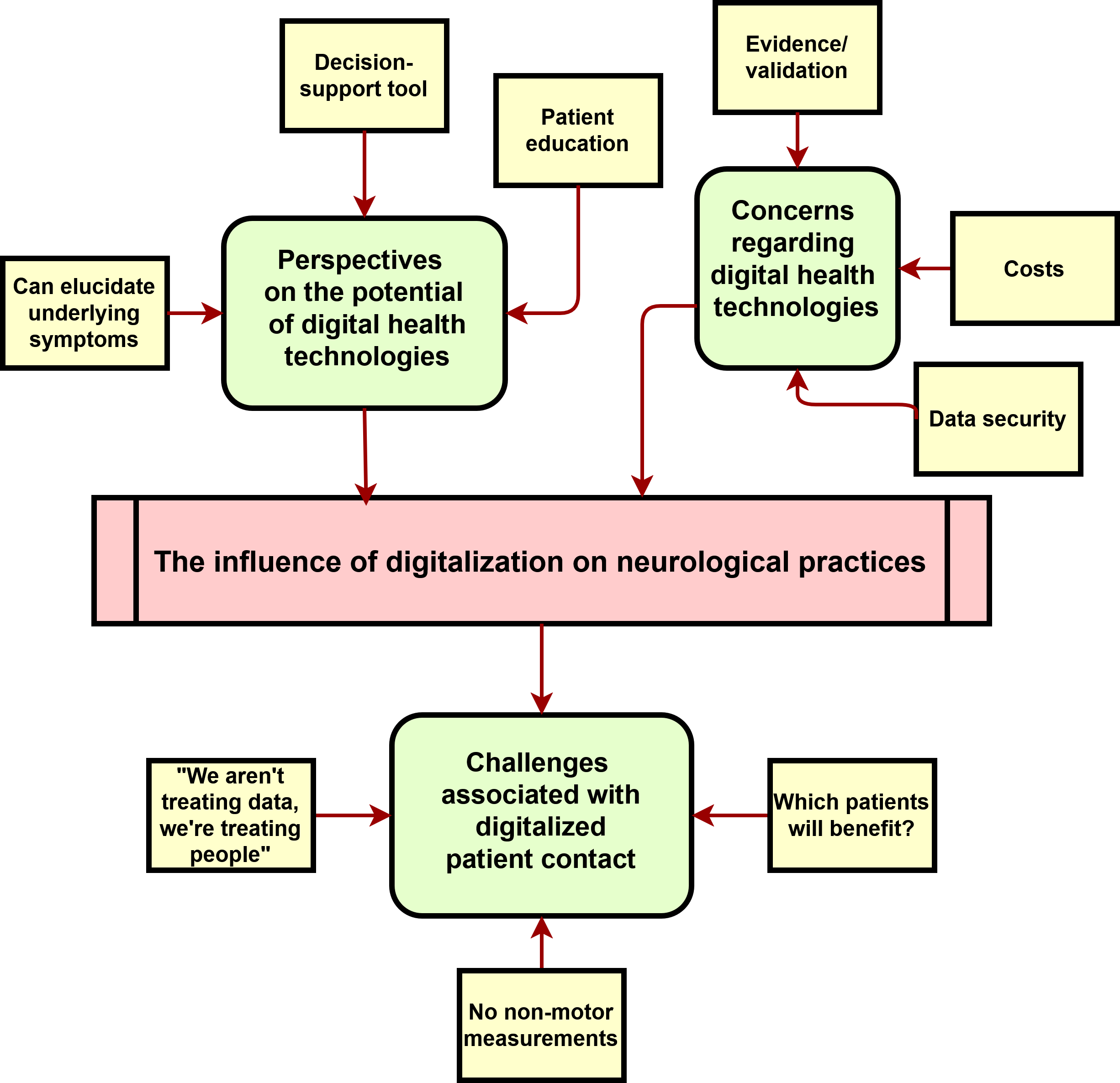

Supplement: Supplementary file 3 [file Image_1.PNG]
